# Dilemma of Adolescent Hypertension

**DOI:** 10.4103/0970-0218.39253

**Published:** 2008-01

**Authors:** Indranil Saha, Bobby Paul, Deepak Kumar Raut

**Affiliations:** Department of Community Medicine, R G Kar Medical College and Hospital, Kolkata, India. E-mail: dr_sahaindranil@rediffmail.com; 1 Department of Community Medicine, Institute of Post Graduate, Medical Education and Research, Kolkata, India.; 2 Department of Epidemiology, All India Institute of Hygiene and Public Health, Kolkata, India.

Sir,

WHO has classified adolescent age group as 10-19 years.(1) Paradoxically during classification of adolescent hypertension, WHO has considered 10-18 years as adolescent and 18 years and above as adult and has given hypertension criteria separately. The purpose of classification of hypertension is to provide an easy and reliable method for characterization of each patient. It allows assessment of severity of disease by reference to epidemiological data so that risk can be defined and appropriate treatment instituted.

Adolescent hypertension has been defined as an average systolic and / or diastolic blood pressure equal to or greater than the 95^th^ percentile for age on at least three occasions (WHO).(2,3) National High Blood Pressure Education Programme (NHBPEP) Coordinating Committee has defined adolescent hypertension as blood pressure that is, on repeated measurement, at the 95^th^ percentile or greater adjusted for age, height and gender.(4) Adult hypertension has been defined as systolic blood pressure of 140 mm Hg or above or a level of diastolic blood pressure of 90 mm Hg or above, in the age group 18 years and above, by WHO.(5) Here lies the contradiction! According to WHO, ‘18 years age group’ is an adolescent by definition, but considered as adult during classification of hypertension.

Moreover in updated Task Force Report on high blood pressure in children and adolescents, National High Blood Pressure Education Programme (NHBPEP) Coordinating Committee has given hypertension criteria for 1-17 years of age.(4)

Hence if one wishes to conduct a survey strictly on ‘adolescent hypertension’ what age group the researcher should select?
